# Post‐Thrombectomy Mild Hypercapnia State Prevents Poor Outcome by Reducing Infarct Progression

**DOI:** 10.1002/brb3.70347

**Published:** 2025-02-28

**Authors:** Peng Jiang, Weitao Yu, Xu Wang, Shouxuan Gao, Yu Geng, Shunyuan Guo, Qiang shen, Longting Lin, Mark Parsons, Sheng Zhang

**Affiliations:** ^1^ Center for Rehabilitation Medicine, Department of Neurology Zhejiang Provincial People's Hospital (Affiliated People's Hospital), Hangzhou Medical College Hangzhou China; ^2^ The Second School of Clinical Medicine Hangzhou Normal University Hangzhou China; ^3^ Center for Rehabilitation Medicine, Department of Radiology Zhejiang Provincial People's Hospital (Affiliated People's Hospital), Hangzhou Medical College Hangzhou China; ^4^ School of Medicine and Public Health University of Newcastle Newcastle New South Wales Australia; ^5^ Department of Neurology Liverpool Hospital University of New South Wales Sydney New South Wales Australia; ^6^ South Western Sydney Clinical School Sydney New South Wales Australia; ^7^ The Ingham Institute for Applied Medical Research Sydney New South Wales Australia

**Keywords:** endovascular therapy, futile recanalization, infarct progress, mild hypercapnia, stroke prognosis

## Abstract

**Objective:**

In endovascular therapy (EVT) for stroke, futile recanalization contributes to poor prognosis. Mild hypercapnia may enhance cerebral blood flow and prevent ischemia, but its impact on prognosis in successfully recanalized EVT patients is unclear.

**Methods:**

We retrospectively analyzed 237 patients from the INSPIRE database who underwent successful recanalization at our center (October 2018–March 2022). Patients were grouped by post‐EVT PaCO_2_ levels: high (40–50 mmHg) and low (<40 mmHg). Significant infarct expansion (SIE) was defined as a decrease in ASPECTS or pc‐ASPECTS by ≥2 from initial to 3–5 days post‐EVT. Poor outcome was modified Rankin Scale score 3–6 at 90 days.

**Results:**

High PaCO_2_ was negatively associated with SIE (OR 0.42, 95% CI 0.22 to 0.84) and poor outcome (OR 0.42, 95% CI 0.20 to 0.87). Mediation analysis showed a significant total effect of high PaCO_2_ on poor outcome (coefficient −0.192, 95% CI −0.345 to −0.046), including an indirect effect mediated by SIE (coefficient −0.055, 95% CI −0.10 to −0.006). These associations were consistent in anterior circulation stroke and patients without severe low PaCO_2_ (<30 mmHg).

**Conclusion:**

Maintaining mild hypercapnia (PaCO_2_ 40–50 mmHg) after EVT may prevent poor outcomes by reducing post‐EVT infarct progression.

## Graphical abstract

Maintaining mild hypercapnia (PaCO_2_ 40–50 mmHg) within the initial 24 h after endovascular therapy may prevent poor outcomes by reducing post‐EVT infarct progression.

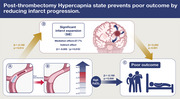

## 1 Introduction

Stroke stands as a leading cause of death and disability globally, particularly in China (GBD 2016 Lifetime Risk of Stroke Collaborators 2018; Wu et al. 2019). Emergency endovascular treatment (EVT) represents a crucial intervention to swiftly restore blood flow by opening blocked large vessels, thereby maximizing the rescue of ischemic brain tissue and significantly improving stroke prognosis (Goyal et al. 2016). Despite achieving more than 70% recanalization rate through EVT (Goyal et al. 2016; Texakalidis et al. 2020), some patients encounter poor outcomes even after successful recanalization, a phenomenon termed futile recanalization (FR), with an estimated incidence ranging from 32.4% to 69.6% (Wang and Xiong 2023).

Research indicates that inadequate restoration of microcirculatory blood flow, especially due to microvascular constriction impeding red blood cell delivery, is a primary mechanism behind FR, significantly impacting the survival of ischemic tissue post‐reperfusion therapy (Dalkara and Arsava 2012). It is well‐established that blood carbon dioxide (CO_2_) plays a crucial regulatory role in cerebral blood flow regulation after ischemic stroke, including microcirculation regulation (Hoiland et al. 2019). Elevated CO_2_ levels have been shown to induce peripheral cell relaxation, improving microcirculatory blood supply and enhancing tissue survival (Matsugi et al. 1997; Mapelli et al. 2017; Hartmann et al. 2021). This may have more significant implications for ischemic tissue perfusion than perioperative peripheral blood pressure levels, which have been extensively studied in the context of EVT (Katsanos et al. 2022). Studies on subarachnoid hemorrhage or post‐cardiac arrest resuscitation suggest that hypercapnia can increase cerebral blood flow and prevent cerebral ischemia (Westermaier et al. 2016; Hope Kilgannon et al. 2019). However, excessively high partial pressure of arterial carbon dioxide (PaCO_2_) levels may elevate the risk of cerebral edema and secondary brain injury (Darkwah Oppong et al. 2021; Cai et al. 2022). Previous studies have associated high PaCO_2_ levels (exceeding 50 mmHg, one of the criteria for type 2 respiratory failure) with poor outcomes in patients with cerebral injury (Cai et al. 2022). Therefore, we speculated that mild hypercapnia (no more than 50 mmHg) might be a pivotal factor influencing stroke prognosis. Nevertheless, research on the relationship between post‐EVT PaCO_2_ levels and clinical prognosis in EVT patients is scarce.

In this study, we hypothesized that post‐EVT mild hypercapnia could reduce the likelihood of poor outcomes by enhancing tissue survival. We prospectively observed the efficacy and safety outcomes in two cohorts of patients who achieved successful recanalization through EVT, including the high PaCO_2_ cohort (PaCO_2_ ≥ 40 mmHg but ≤ 50 mmHg) and the low PaCO_2_ cohort (PaCO_2 _< 40 mmHg), aiming to elucidate the relationship between mild hypercapnia, ischemic tissue survival, and stroke prognosis after EVT.

## 2 Methods

### 2.1 Ethics Statement

All patients or appropriate family members gave written informed consent before the study. The protocols of the study were approved by the Human Ethics Committee of Zhejiang Provincial People's Hospital (No. 2017KY021). All clinical investigations were conducted according to the principles set in the Declaration of Helsinki.

### 2.2 Patient Selection

We reviewed our prospectively collected data of patients with acute large vessel occlusion (LVO) who were admitted within 24 h after stroke onset, which were registered into the International Stroke Perfusion Imaging Registry (INSPIRE) database (Lin et al. 2023) from October 2018 to March 2022. Patients were included if (i) they achieved successful recanalization after EVT; (ii) they had undergone blood gas analysis monitoring at least twice within the first 24 h after EVT with a minimum interval of 4 h; (iii) their mean PaCO_2_ < 50 mmHg and mean partial pressure of arterial oxygen (PaO_2_) ≥ 60 mmHg in the first 24 h; (iv) the previous modified Rankin Scale (mRS) scores < 2. Patients were excluded if: (i) they had incomplete image data post‐EVT; (ii) they had advanced‐stage cancer with brain metastasis, severe intracranial infection, severe decompensated kidney, or heart failure; (iii) brain vascular imaging (computed tomography angiography [CTA]/magnetic resonance angiography [MRA]) confirmed the re‐occlusion of the recanalized vessel after EVT; (iv) they were lost to follow‐up.

Eligible patients received intravenous thrombolysis prior to EVT. In routine clinical practice, EVT is offered to patients with (i) the presence of an LVO that is potentially retrievable, (ii) reasonable pre‐stroke function (mRS 0–2), and (iii) limited medical comorbidity. The National Institute of Health Stroke Severity (NIHSS), the primary index for stroke severity, was assessed immediately before treatment and 24‐h imaging. All patients were assessed at 90 days post‐stroke onset for the functional outcome using the mRS.

### 2.3 The Calculation of PaCO_2_ Level

We utilized the ABL800 FLEX Blood gas, oximetry, electrolyte, and metabolite analyzer to perform blood gas analysis. Patients underwent blood gas analysis monitoring at least twice within the first 24 h after EVT with a minimum interval of 4 h. The level of PaCO_2_ was calculated to take the average of PaCO_2_ values.

Based on our study design, patients were divided into two cohorts according to the average PaCO_2_ level within the first 24 h after EVT: the high PaCO_2_ group (PaCO_2_ ≥ 40 mmHg but < 50 mmHg) and the low PaCO_2_ group (PaCO_2_ < 40 mmHg).

### 2.4 Imaging Protocols

All patients underwent baseline noncontrast computed tomography (NCCT) and computed tomography perfusion imaging (CTP), 24‐h NCCT after EVT, and 3–5 days NCCT or MRI after EVT. All computed tomography (CT) imaging scans were acquired on a 320‐detector row 640‐slice cone beam multi‐detector row CT (MDCT) scanner (Aquilion One, Toshiba Medical Systems). Whole‐brain NCCT was performed in one rotation (detector width, 16 cm). After NCCT, a CTP was acquired after administration of 50 mL of a contrast agent (Ultravist 370; Bayer HealthCare, Berlin, Germany) injected intravenously at a rate of 6 mL/s chased by 50 mL of saline (acquisition parameters: 120 kV, 128 mAs; scanning coverage = 240 mm, scanning width = 5 mm). Starting 7 s after contrast injection, a pulsed full‐rotation scan with 18‐time points acquired over 60 s with a total pulse image acquisition time of 9.5 s was used. The scanning protocol of follow‐up NCCT was the same as that of baseline NCCT. All cranial magnetic resonance imagings (MRIs) were conducted using a 3.0T magnetic resonance (MR) scanner. The sequences included T1‐weighted imaging repetition time or time of repetition (TR) / echo time or time of echo (TE): 160/3.05 ms), T2‐weighted imaging (TR/TE: 6000/100 ms), T2‐fluid‐attenuated inversion recovery (FLAIR) imaging (TR/TE: 9000/94.0 ms), and diffusion‐weighted imaging (DWI) (TR/TE: 6400/86.0 ms, *b* value of 0 and 1000 s/mm^2^). The slice thickness and spacing were set at 5 and 1.5 mm, respectively.

### 2.5 Imaging Measurement

#### 2.5.1 Recanalization Status

Digital subtraction angiography (DSA) images were acquired using the FD20 DSA imaging system from Philips Healthcare. The pre‐ and post‐procedural angiographic findings were evaluated based on the Modified Thrombolysis in Cerebral Infarction Score (mTICI) (Zaidat et al. 2013). Successful recanalization was defined as mTICI 2b‐3 after the procedure.

## 3 ASPECTS

The Acute Stroke Prognosis Early CT Score (ASPECTS) was determined based on the extent of low density on CT scans. It is divided into 10 regions, with 1 point subtracted for each region showing early ischemic changes such as focal swelling or parenchymal hypoattenuation. The maximum score is 10, while the minimum score is 0 (Barber et al. 2000). For posterior circulation stroke, the posterior circulation ASPECTS (pc‐ASPECTS) was used, allocating 10 points for the posterior circulation (Puetz et al. 2008). In very few cases where NCCT was not available, follow‐up MR DWI hyperintense signal was used, requiring a confluent area of hyperintensity rather than small specks (Desilles et al. 2017).

To study the status of ischemic tissue survival, ASPECTS or pc‐ASPECTS reduction (pre‐EVT ASPECTS minus post‐EVT ASPECTS which was based on 3–5 days NCCT after EVT) was used for a semi‐quantitative analysis of post‐EVT infarct volume expansion. Significant infarct expansion (SIE) was defined as ASPECTS or pc‐ASPECTS reduction ≥ 2 points. As a matter of convenience, we hereinafter used ASPECTS uniformly refer to ASPECTS and pc‐ASPECTS, and ASPECTS reduction uniformly refer to ASPECTS reduction and pc‐ASPECTS reduction.

The images were independently reviewed by two trained neurologists (P.J. and SY.G., with over 5 years of experience in neuroimaging research) who were blinded to patient outcomes. For any discrepancy between the two readers, another neurologist of higher seniority (S.Z.) re‐evaluated the images and helped to reach a consensus.

### 3.1 Clinical Outcomes

Poor outcome was defined as a modified Rankin Scale (mRS) score of 3 to 6 at 90 days after the onset of stroke. The safety outcomes included mortality within 90 days and symptomatic intracerebral hemorrhage (sICH), defined as parenchymal hematoma type 2 (defined as clots in at least 30% of the infarcted area with space‐occupying effect) within 48 h after EVT in combination with worsening of NIHSS score by at least 4 points, or from the lowest NIHSS value between baseline and 24 h, or leading to death (Wahlgren et al. 2007).

### 3.2 Statistical Analyses

Statistical analyses were performed using adjusted logistic regression models to estimate odds ratios (ORs) with corresponding two‐sided 95% confidence intervals (CIs) for poor outcomes at 90 days, considering potential confounders that were not fully balanced, with a prespecified *p*‐value of < 0.05 for baseline comparisons between groups. Baseline data were analyzed using Student's *t*‐test (presented as mean ± standard deviation) or Mann‐Whitney U test (presented as median and range) for continuous data, depending on normality assumptions. Categorical variables were presented as numbers and percentages, and comparisons were made using the chi‐square test. Binary logistic regression analysis was used to analyze the association among PaCO_2_ level, SIE, and 90‐day poor outcomes. The possible mediation effect of SIE between PaCO_2_ and poor outcome was tested using a mediation effect analysis.

Considering previous studies suggesting potential harm from significantly reduced PaCO_2_ levels in brain injury‐related diseases (Cai et al. 2022), the above analyses were repeated after excluding extremely low PaCO_2_ patients (PaCO_2 _< 30 mmHg). We also made subgroup analysis in anterior and posterior‐circulation stroke patients, separately. Statistical testing of outcomes was conducted at a two‐tailed *α* level of 0.05. Statistical analyses were performed using SPSS 25.0 software and Empower (R) (www.empowerstats.com, X&Y solution, Inc., Boston, MA) and GraphPad Prism software (Version 8.0.2).

## 4 Results

### 4.1 Population Description

**FIGURE 1:**
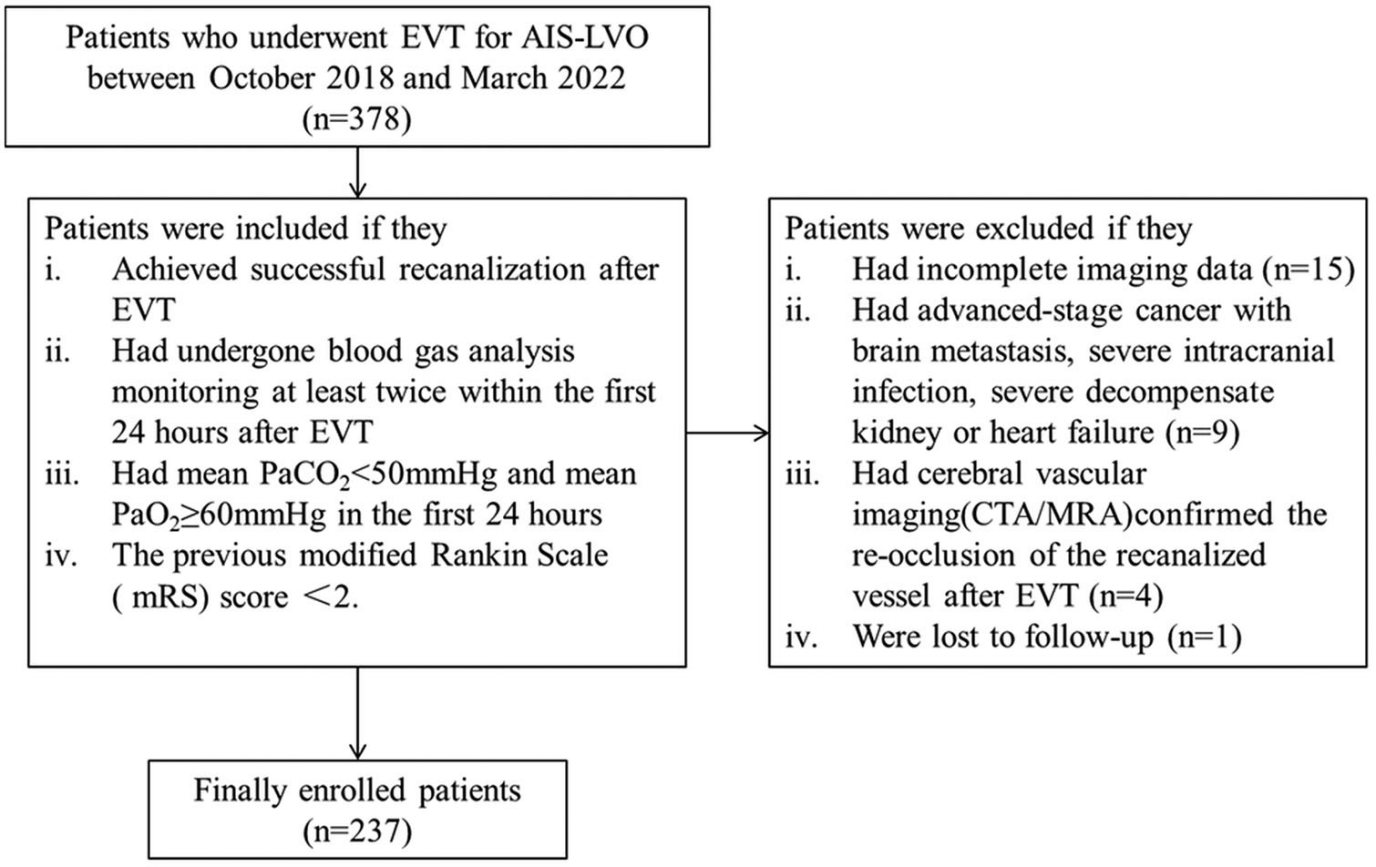
**Patient selection flowchart**. AIS‐LVO, acute ischemic stroke with large vessel occlusion; CTA, computed tomography angiography; EVT, endovascular treatment; MRA, magnetic resonance angiography; mRS, modified Rankin Scale; PaCO_2_, partial pressure of arterial carbon dioxide; PaO_2_, partial pressure of arterial oxygen.

A total of 378 patients with LVO who underwent EVT were initially considered. Following the predefined inclusion and exclusion criteria, 237 patients who achieved successful recanalization were ultimately included in our analysis. The patient selection process is illustrated in Figure 1. Among all patients (*n* = 237), the median age was 70y (IQR 60–79y), the median NIHSS score was 18 (IQR 14–24), the median baseline ASPECTS was 8 (IQR 6–9), and the median ASPECTS reduction was 2 (1–4). Bridging therapy was performed in 85 patients (35.9%). At 90 days, 151 out of 237 patients (63.7%) experienced poor outcomes.

Characteristics of all patients in the final analysis are presented in Table 1. Among them (*n* = 237), 45 were classified into the high PaCO_2_ group, and 192 into the low PaCO_2_ group. In comparison to the high PaCO_2_ group, the low PaCO_2_ group exhibited a younger age (median: 65 vs. 71 years, *p =* 0.012), a lower proportion of females (8.9% vs. 45.8%, *p < *0.001), lower prevalence of comorbid atrial fibrillation (22.2% vs. 51.0%, *p < *0.001), and rt‐PA administration (24.4% vs. 38.5%, *p =* 0.076), a higher rate of smoking (44.4% vs. 13.5%, *p < *0.001), and a higher PaO_2_ level (median: 200.0 vs. 166.5, *p =* 0.033) (Table 1).

**TABLE 1:** Comparisons for baseline characteristics and outcomes between two PaCO_2_ cohorts.

Characteristic	PaCO_2_ cohorts			*p*‐value ^b^
	Overall, *n* = 237^a^	Low PaCO_2_ *n* = 192^a^	High PaCO_2_ *n* = 45^a^	
Age, median (IQR)	70 (60, 79)	71 (61, 80)	65 (57, 74)	0.013
Female, *n* (%)	92 (38.8%)	88 (45.8%)	4 (8.9%)	<0.001
**Risk factors**, *n* (%)				
Hypertension	152 (64.1%)	126 (65.6%)	26 (57.8%)	0.323
Diabetes	37 (15.6%)	33 (17.2%)	4 (8.9%)	0.167
AF	108 (45.6%)	98 (51.0%)	10 (22.2%)	<0.001
Smoking	46 (19.4%)	26 (13.5%)	20 (44.4%)	<0.001
Stroke	26 (11.0%)	22 (11.5%)	4 (8.9%)	0.793
**Baseline characteristics**				
PaCO_2_, median (IQR)	35.4 (32.0, 38.CC7)	34.4 (31.3, 36.9)	42.2 (41.1, 44.7)	<0.001
PaO_2_, median (IQR)	168 (137, 210)	166 (136, 203)	200 (148, 228)	0.033
pH, median (IQR)	7.39 (7.37, 7.43)	7.40 (7.39, 7.43)	7.37 (7.35, 7.38)	<0.001
NIHSS, median (IQR)	18 (14, 24)	18 (14, 24)	18 (13, 21)	0.375
Ischemic core volume, mL, median (IQR)	21 (6, 47)	22 (7, 49)	18 (6, 35)	0.653
Penumbra volume, mL, median (IQR)	91 (58, 129)	91 (57, 130)	91 (62, 129)	0.822
Posterior circulation stroke, *n* (%)	45 (19.0%)	40 (20.8%)	5 (11.1%)	0.134
SBP, mmHg, median (IQR)	155 (139, 170)	156 (141, 171)	149 (134, 169)	0.153
DBP, mmHg, median (IQR)	88 (77, 101)	89 (78, 101)	85 (77, 95)	0.260
PP mmHg, median (IQR)	66 (52, 80)	67 (52, 80)	63 (54, 76)	0.601
PLT, 10^9^/L, median (IQR)	176 (138, 206)	177 (131, 205)	174 (147, 222)	0.384
Intravenous thrombolysis, *n* (%)	85 (35.9%)	74 (38.5%)	11 (24.4%)	0.076
Angioplasty	63 (26.6%)	48 (25.0%)	15 (33.3%)	0.255
**Outcome**				
ASPECTS reduction, points, median (IQR)	2.00 (1.00, 4.00)	3.00 (1.00, 4.00)	2.00 (1.00, 3.00)	0.021
SIE, *n* (%)	74 (31.2%)	52 (27.1%)	22 (48.9%)	0.004
3m‐mRS score, median (IQR)	3.00 (2.00, 5.00)	4.00 (2.00, 5.00)	2.00 (1.00, 4.00)	0.001
Poor outcome, *n* (%)	151 (63.7%)	132 (68.8%)	19 (42.2%)	<0.001
3‐month mortality, *n* (%)	38 (16.0%)	35 (18.2%)	3 (6.7%)	0.057
sICH, *n* (%)	24 (10.1%)	21 (10.9%)	3 (6.7%)	0.583

Abbreviations: AF, atrial fibrillation; ASPECTS, Alberta Stroke Program Early CT Score; DBP, diastolic blood pressure; IQR, interquartile range; mRS, modified Rankin Score; NIHSS, National Institutes of Health Stroke Scale; PaCO_2_, partial pressure of arterial carbon dioxide; PaO_2_, partial pressure of arterial oxygen; pH, potential of hydrogen; PLT, platelet; PP, pulse pressure; SBP, systolic blood pressure; sICH, symptomatic intracerebral hemorrhage.; SIE, significant infarct expansion.

^a^ Median (IQR); n (%).

^b^ Wilcoxon rank sum test; Pearson's Chi‐squared test; Fisher's exact test.

### 4.2 The Association Between PaCO_2_ Status and 90‐Day Poor Outcome

**FIGURE 2:**
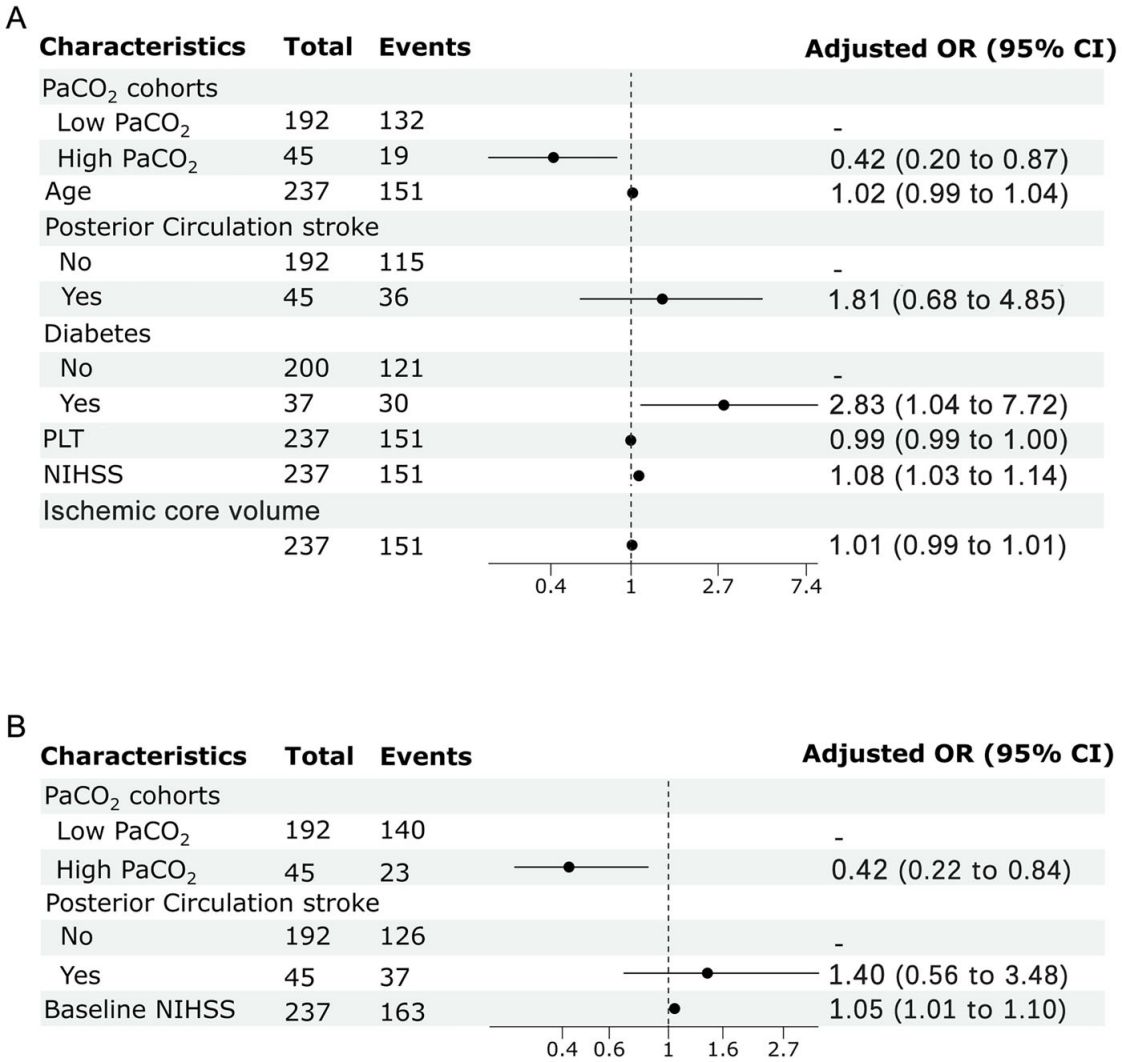
Multivariable logistic regression analysis for the association of PaCO_2_ status with 90‐day poor outcomes (A) and SIE (B). NIHSS, National Institutes of Health Stroke Scale; PLT, blood platelet.

Overall, 151 patients (63.7%) experienced a poor outcome at the 3‐month follow‐up. Patients with high PaCO_2_ demonstrated a significantly lower rate of poor outcomes compared to those with low PaCO_2_ (68.8% vs. 42.2%, *p < *0.001). Further details regarding the comparison between patients with good and poor outcomes are provided in Table S1. Multivariate regression analysis, adjusting for age, posterior circulation, diabetes, platelet count (PLT), ischemic core volume, and baseline NIHSS score, revealed a negative association between high PaCO_2_ and poor outcome (OR 0.42, 95% CI 0.20 to 0.87) (Figure 2A).

### 4.3 The Association Between PaCO_2_ Status and SIE

In the entire patient cohort (*n* = 237), the high PaCO_2_ group, in comparison to the low PaCO_2_ group, exhibited a lower ASPECTS reduction (median: 2 vs. 3, *p =* 0.021) and a decreased incidence of SIE (30% vs. 70%, *p =* 0.004) post‐EVT (Table 1). Table S2 provides additional details regarding the comparison between SIE and non‐SIE patients. Multivariable regression analysis, adjusting for posterior circulation stroke and baseline NIHSS score, revealed a negative association between high PaCO_2_ and SIE (OR 0.42, 95% CI 0.22 to 0.84) (Figure 2B).

### 4.4 Mediation Analysis

**FIGURE 3:**
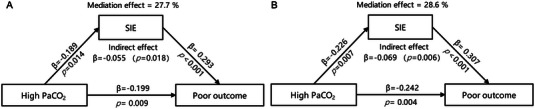
Mediation effect of SIE on the association between high PaCO_2_ and poor outcome in overall patients enrolled (A) and in anterior circulation subgroup (B). SIE, significant infarct expansion.

Mediation analysis showed that the total effect of PaCO_2_ levels on poor outcomes was statistically significant with a coefficient of −0.199 (95% CI −0.347 to −0.052, *p =* 0.009). The indirect effect mediated by SIE was also statistically significant with a coefficient of −0.055 (95% CI −0.10 to −0.006, *p =* 0.018). The direct effect of high PaCO_2_ on the poor outcome, after controlling for SIE, was still statistically significant with a coefficient of −0.144 (95% CI −0.287 to −0.001, *p =* 0.049). The proportion of the total effect mediated by SIE was 27.7% (Figure 3A).

### 4.5 Safety Outcomes

The high PaCO_2_ group exhibited a lower 90‐day mortality rate compared to the low PaCO_2_ group, although the difference was not statistically significant (6.7% vs. 18.2%, *p =* 0.057). Similarly, rates of sICH did not differ significantly between the two groups (6.7% vs. 10.9%, *p =* 0.562). Univariate and multivariate regression analyses demonstrated that PaCO_2_ status was not associated with sICH or 90‐day mortality (Tables S3 and S4). These findings remained consistent after adjusting for stroke location and excluding patients with severely low PaCO_2_ levels (all *p > *0.05).

### 4.6 Excluding Patients With Severe Low PaCO_2_ Level

**FIGURE 4:**
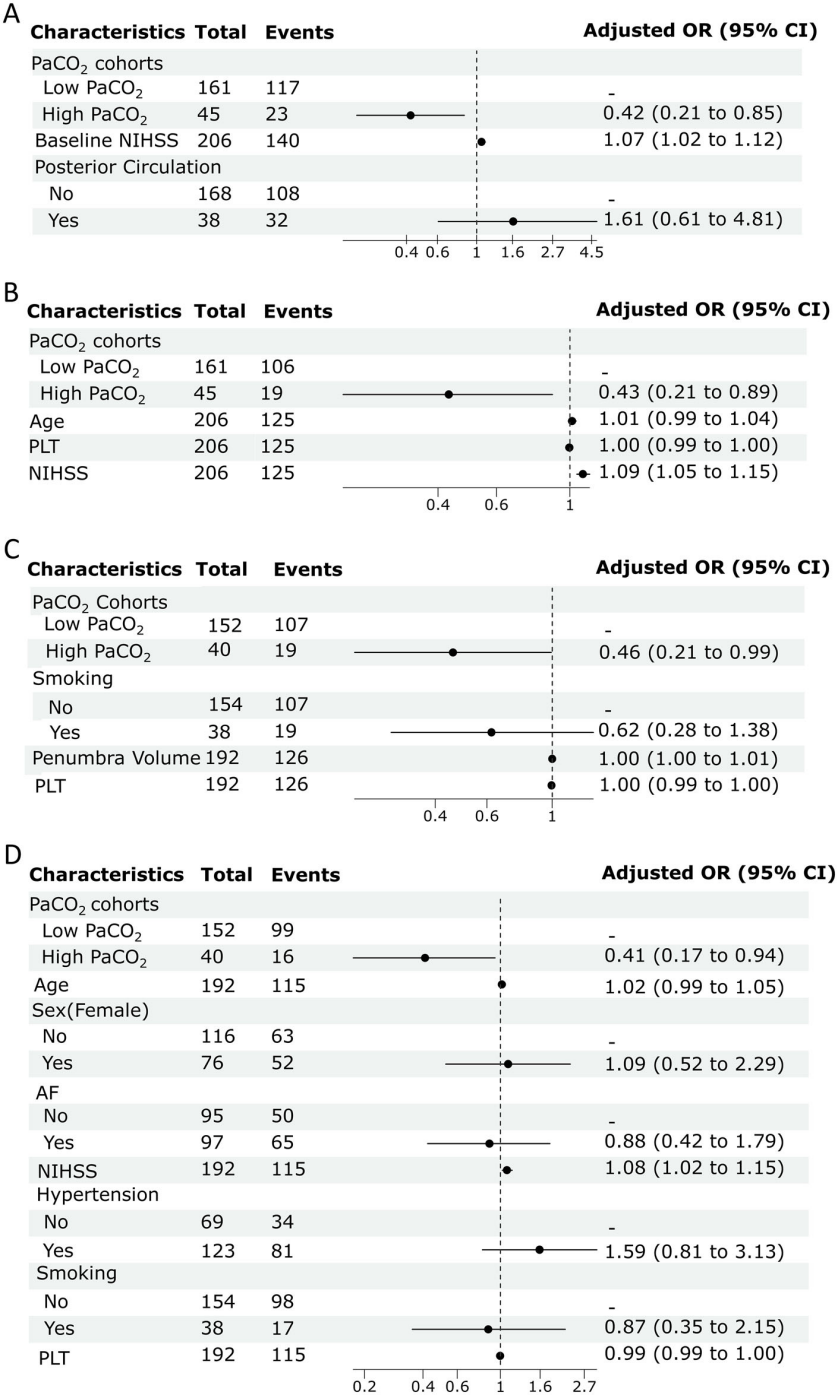
Multivariable logistic regression for the association of PaCO_2_ status with 90‐day poor outcomes (A) and SIE (B) after excluding patients with severe low PaCO_2_ level. Multivariable logistic regression for the association of PaCO_2_ status with SIE (C) and 90‐day poor outcomes (D) in the anterior circulation stroke subgroup. AF, atrial fibrillation; NIHSS, National Institutes of Health Stroke Scale; PLT, platelet.

After excluding extremely low PaCO_2_ patients (PaCO_2 _< 30 mmHg) (*n* = 31), high PaCO_2_ was still negatively associated with SIE (OR 0.42, 95% CI 0.21 to 0.85) and poor outcome (OR 0.43, 95% CI 0.21 to 0.89, *p < *0.01) (Figure 4A,B). The total effect of high PaCO_2_ on poor outcomes was also significant (coefficient −0.17580, 95% CI −0.335 to −0.029, *p =* 0.008). The indirect effect, mediated by SIE, was also significant (coefficient −0.050, 95% CI −0.110 to −0.001, *p =* 0.044), suggesting a mediating role of SIE in the relationship. The direct effect of PaCO_2_ levels on poor outcomes was marginally non‐significant (coefficient −0.126, 95% CI −0.287 to 0.013, *p =* 0.088). The proportion mediated by SIE was 28.9%.

### 4.7 Anterior Circulation Versus Posterior Circulation Stroke Subgroup Analysis

Based on the distribution of stroke location, all patients (*n* = 237) were categorized into two subgroups: anterior circulation stroke (*n* = 192) and posterior circulation stroke (*n* = 45). We observed a significant difference in PaCO_2_ levels between anterior and posterior circulation stroke patients (35.7 ± 4.9 vs. 34.1 ± 4.3 mmHg, *p =* 0.013) (Table S5).

In the anterior circulation stroke subgroup, 40 patients had PaCO_2_ ≥ 40 mmHg, with 16 (40.0%) experiencing a poor outcome at 90 days. Among the 152 patients with PaCO_2 _< 40 mmHg, 99 (65.1%) had a poor outcome at 90 days. In the posterior circulation stroke subgroup, 5 patients had PaCO_2_ ≥ 40 mmHg, with 3 (60.0%) having a poor outcome at 90 days. Among the 40 patients with PaCO_2 _< 40 mmHg, 33 (82.5%) had a poor outcome at 90 days. Multivariate regression analysis in the anterior circulation stroke subgroup indicated that high PaCO_2_ was associated with SIE (OR 0.46, 95% CI 0.21 to 0.99, *p =* 0.047) and poor outcomes (OR 0.41, 95% CI 0.17 to 0.94, *p =* 0.036) after adjusting for co‐factors (Figure 4C,D). However, no such relationship was found in the posterior circulation stroke subgroup. PaCO_2_ status was not associated with sICH and 90‐day mortality in either the anterior or posterior circulation stroke subgroups (all *p > *0.05).

In anterior circulation stroke, the total effect of high PaCO_2_ on poor outcomes was found to be significant (coefficient −0.242, 95% CI −0.405 to −0.080, *p =* 0.004). The indirect effect, mediated by SIE, was also significant (coefficient −0.069, 95% CI −0.113 to −0.013, *p =* 0.006), indicating a mediating role of SIE in the relationship. In addition, the direct effect of high PaCO_2_ on poor outcomes remained significant (coefficient −0.173, 95% CI −0.331to −0.015, *p =* 0.033). The proportion mediated by the ASPECTS score was 28.6% (Figure 3B).

## 5 Discussion

This study reveals that maintaining a mild hypercapnia state (PaCO_2_ levels of 40–50 mmHg) within the first 24 h after successful recanalization in EVT is associated with a reduced risk of SIE and improved outcomes at 90 days. Subgroup analyses affirm the robustness of these associations in patients with anterior circulation stroke and those excluding severe low PaCO_2_ levels. Furthermore, our findings suggest that SIE partially mediates the relationship between elevated PaCO_2_ and improved outcomes in EVT patients.

In this study, we found the effect of a mild hypercapnia state in avoiding FR and poor outcomes. The possible mechanism for these associations can be explained as follows. It is known that the key of FR was microvessel constriction (Dalkara and Arsava 2012), and the contractility of pericytes is the basis of microvascular blood flow regulation (Hall et al. 2014; Liu et al. 2020). Yemisci et al. (2009) discovered that the pathologically sustainably constricted microvessel segments, despite successful reopening of the middle cerebral artery, sustained contraction leads to segmental capillary stenosis, which blocks red blood cell delivery and leads to a breakdown of microcirculation, accounts for the heterogeneous acidity in the penumbra area due to mismatch between oxygen and glucose supply by hindering passage of blood cells and promoting their aggregation together with fibrin while allowing plasma flow, and is likely to be crucial in determining the infarct tissue fate (Hossmann 2006). Nevertheless, this microvessel constriction caused primarily by pericytes has also been shown to be reversible (Yemisci et al. 2009). CO_2_ is one of the strongest regulators of cerebral blood flow. Even small changes in PaCO_2_ can result in significant changes in cerebral blood flow, including microcirculation blood flow, with approximately 3%–8% change for every 1 mmHg change in PaCO_2_ (Hoiland et al. 2019). Hartmann et al. (2021) also found that pericytes in capillaries can expand and contract slowly under the induction of hypercapnia. Our results also support these previous findings. We further proved that PaCO_2_ was negatively associated with poor outcomes to some extent, which was mainly mediated by the effect that mild hypercapnia prevented infarct progression. In addition, considering the harm of excessively high or low PaCO_2_ levels (Darkwah Oppong et al. 2021; Cai et al. 2022; Curley et al. 2010; Solaiman and Singh 2013), we found an effective and safe range of PaCO_2_ (40–50 mmHg) that can be applied to avoid FR and poor outcome, which should be validated in further large sample sized prospective studies.

In this study, no correlation was found between PaCO_2_ level and stroke prognosis in the posterior circulation stroke, this may be attributed to the small sample size. Our univariate analysis found that the PaCO_2_ level in posterior circulation stroke was relatively lower, and the rate of severely low PaCO_2_ level was higher than that in anterior circulation stroke. Therefore, we speculated that patients with posterior circulation stroke were more likely to have poor outcomes than patients with anterior circulation stroke as it is now believed that hypocapnia did more harm than benefit for brain injury (Curley et al. 2010). However, due to the lack of a sufficient number of patients with high PaCO_2_ in the posterior circulation stroke subgroup, the analysis was unable to further prove the impact of PaCO_2_ on stroke outcome. Besides, it is unclear whether a relatively low PaCO_2_ was presented in posterior circulation stroke before EVT or just the result of self‐regulation of cerebral vessels after thrombus retrieval. Further research is needed to clarify what factors influence the PaCO_2_ level after EVT and its mechanism.

Of note, our study revealed that approximately one‐third of patients (31.2%) experienced SIE after successful EVT, indicating that FR is not uncommon in this context. By examining blood samples, we identified PaCO_2_ levels as an intervenable marker that can predict the occurrence of FR. This holds promising implications for improving stroke outcomes and potentially reducing FR in the future.

This study has several limitations. First, it is a retrospective study, which makes it difficult to establish clear causal relationships and is prone to patient selection biases. Second, our study included individuals with PaCO_2_ levels below 50 mmHg. Previous research has suggested that PaCO_2_ levels exceeding type 2 respiratory failure criteria may be significantly harmful (Cai et al. 2022). As a retrospective study, it is rare for clinicians to allow patients to maintain PaCO_2_ levels above 50 mmHg without intervention. However, considering the results of our study, it is worth questioning whether a prospective interventional study should adopt a range of PaCO_2_ values greater than 50 mmHg in a short time after EVT. Third, all the patients included in our study were under general anesthesia. It is uncertain whether the results obtained are applicable to patients under local anesthesia. However, previous studies have suggested that anesthesia does not have a significant impact on cerebral vasoreactivity to CO_2_ (Grüne et al. 2015). Fourth, the consistency is not high in ASPECTS. However, Gerschenfeld et al. (2017) suggested that there was no difference in the accuracy of CT perfusion and ASPECTS for predicting the volume of MRI lesions in the hyperacute phase of acute ischemic stroke. Previous studies have shown that in patients undergoing EVT, there were no significant differences in the clinical outcomes of patients selected with NCCT compared with those selected with CTP or MRI whether within 6 h (Jadhav et al. 2022) or 6–24 h (Nguyen et al. 2022). As a simple and easy method, ASPECTS has been widely used in the field of EVT for acute ischemic stroke.

## 6 Conclusion

In conclusion, maintaining a mild hypercapnia state (PaCO_2_ ranging from 40 to 50 mmHg) within the initial 24 h after EVT for large vessel occlusion (LVO) may prove beneficial in preventing poor outcomes at 90 days by mitigating post‐EVT infarct progression compared to lower PaCO_2_ levels. Importantly, this approach does not pose an increased risk of symptomatic intracranial hemorrhagic transformation or death.

## Author Contributions

Concept and design: P.J. and S.Z.; data collection and analysis: WT.Y., X.W., P.J., SY.G. and S.Z.; drafting of the article: P.J.; assisting the literature search: SX.G., WT.Y. and X.W.; software and technical support: Q.S., LT.L. and M.P.; critical revision of the article: S.Z.; study supervision: Y.G. All authors reviewed the manuscript.

## Conflicts of Interest

The authors declare no conflicts of interest.

### Peer Review

The peer review history for this article is available at https://publons.com/publon/10.1002/brb3.70347

## Supporting information



Supporting Information

## Data Availability

All relevant data during this study are available from the corresponding author on reasonable request.
