# Supplementary material for: Post‐Thrombectomy Mild Hypercapnia State Prevents Poor Outcome by Reducing Infarct Progression
Source: Brain Behav. 2025 Feb 28;15(3):e70347. doi: 10.1002/brb3.70347 (PMC11870790; doi:10.1002/brb3.70347)
Supplement: Supplementary file 1 — Supporting Information [file BRB3-15-e70347-s001.docx]

**Supplementary Table I.** **Comparisons for baseline characteristics and outcomes between patients with good and poor outcome.**

| Variables | Overall, n = 237^1^ | | good outcome  n = 86^1^ | poor outcome  n = 151^1^ | *p*-value |
| --- | --- | --- | --- | --- | --- |
| Age | 70 (60, 79) | | 65 (56, 76) | 72 (63, 80) | 0.003 |
| Female | 92 (39%) | | 27 (31%) | 65 (43%) | 0.077 |
| **Risk factors** |  | |  |  |  |
| Hypertension | 152 (64%) | | 49 (57%) | 103 (68%) | 0.083 |
| Diabetes | | 37 (16%) | 7 (8%) | 30 (20%) | 0.017 |
| AF | | 108 (46%) | 33 (38%) | 75 (50%) | 0.093 |
| Smoking | | 46 (19%) | 22 (26%) | 24 (16%) | 0.070 |
| Stroke | | 26 (11%) | 9 (10%) | 17 (11%) | 0.851 |
| **Baseline characteristics** | |  |  |  |  |
| PaCO_2_ | | 35.7 ± 4.9 | 37.2 ± 4.9 | 34.7 ± 4.6 | <0.001 |
| PaO_2_ | | 168 (137, 210) | 168 (141, 227) | 167 (137, 202) | 0.302 |
| pH | | 7.40 ± 0.04 | 7.39 ± 0.04 | 7.40 ± 0.04 | 0.131 |
| NIHSS | | 18 (14, 24) | 16 (12, 19) | 19 (15, 27) | <0.001 |
| Ischemic core volume | | 21 (6, 47) | 15 (6, 29) | 23 (7, 58) | 0.007 |
| Penumbra volume | | 91 (58, 129) | 84 (50, 123) | 94 (59, 131) | 0.314 |
| Posterior circulation | | 45 (19%) | 9 (10%) | 36 (24%) | 0.012 |
| SBP (mmHg) | | 156 ± 25 | 154 ± 24 | 158 ± 25 | 0.224 |
| DBP (mmHg) | | 88 (77, 101) | 88 (78, 100) | 87 (78, 101) | 0.610 |
| PP (mmHg) | | 67 ± 20 | 64 ± 20 | 69 ± 20 | 0.061 |
| Intravenous thrombolysis | | 85 (36%) | 30 (35%) | 55 (36%) | 0.812 |
| Angioplasty | | 63 (27%) | 24 (28%) | 39 (26%) | 0.728 |
| PLT | | 176 (138, 206) | 187 (146, 215) | 172 (134, 201) | 0.050 |
| **Outcome, n (%)** | |  |  |  |  |
| ASPECTS reduction | | 2.00 (1.00, 4.00) | 1.00 (0.00, 3.00) | 3.00 (2.00, 5.00) | <0.001 |
| SIE | | 163 (69%) | 40 (47%) | 123 (81%) | <0.001 |
| 3-month mRS | | 3.00 (2.00, 5.00) | 1.00 (1.00, 2.00) | 4.00 (4.00, 5.50) | <0.001 |
| 3-month mortality | | 38 (16%) | 0 (0%) | 38 (25%) | <0.001 |
| sICH | | 24 (10%) | 1 (1%) | 23 (15%) | <0.001 |

ASPECTS, alberta stroke program early CT score; PaCO_2_, partial pressure of arterial carbon dioxide; PaO_2_, partial pressure of arterial oxygen; pH, potential of hydrogen; PP, pulse pressure; AF, atrial fibrillation; SBP, systolic blood pressure; DBP, diastolic blood pressure; IQR, interquartile range; mRS, modified rankin score; NIHSS, national institutes of health stroke scale; sICH, symptomatic intracerebral hemorrhage; PLT, platelet. SIE, significant infarct enlargement.

**Supplementary Table II. Comparisons for baseline characteristics and outcomes between SIE and non-SIE patients.**

| Variables | Overall, n = 237^1^ | Non-SIE, n = 74^1^ | SIE, n = 163^1^ | *p*-value |
| --- | --- | --- | --- | --- |
| Age | 70 (60, 79) | 69 (56, 80) | 71 (61, 79) | 0.697 |
| Sex (Female) | 92 (39%) | 26 (35%) | 66 (40%) | 0.433 |
| **Risk factors** |  |  |  |  |
| Hypertension | 152 (64%) | 48 (65%) | 104 (64%) | 0.875 |
| Diabetes | 37 (16%) | 8 (11%) | 29 (18%) | 0.170 |
| AF | 108 (46%) | 31 (42%) | 77 (47%) | 0.444 |
| Smoking | 46 (19%) | 19 (26%) | 27 (17%) | 0.100 |
| Stroke | 26 (11%) | 9 (12%) | 17 (10%) | 0.692 |
| **Baseline characteristics** |  |  |  |  |
| PaCO_2_ | 35.7 ± 4.9 | 36.4 ± 5.4 | 35.3 ± 4.6 | 0.127 |
| High PaCO_2_ | 45 (19%) | 22 (30%) | 23 (14%) | 0.004 |
| PaO_2_ | 168 (137, 210) | 167 (140, 219) | 169 (137, 205) | 0.762 |
| pH | 7.40 ± 0.04 | 7.40 ± 0.04 | 7.40 ± 0.04 | 0.421 |
| NIHSS | 18 (14, 24) | 16 (13, 20) | 19 (14, 25) | 0.004 |
| Ischemic core volume | 21 (6, 47) | 15 (5, 34) | 23 (9, 53) | 0.087 |
| Penumbra volume | 91 (58, 129) | 82 (45, 121) | 94 (60, 130) | 0.186 |
| Posterior circulation | 45 (19%) | 8 (11%) | 37 (23%) | 0.031 |
| SBP (mmHg) | 156 ± 25 | 157 ± 26 | 156 ± 25 | 0.619 |
| DBP (mmHg) | 88 (77, 101) | 89 (80, 100) | 87 (75, 101) | 0.309 |
| PP (mmHg) | 67 ± 20 | 66 ± 21 | 67 ± 19 | 0.878 |
| PLT | 176 (138, 206) | 183 (148, 210) | 172 (132, 205) | 0.184 |
| IV thrombolysis | 85 (36%) | 24 (32%) | 61 (37%) | 0.458 |
| Angioplasty | 63 (27%) | 21 (28%) | 42 (26%) | 0.673 |
| **Outcome, n (%)** |  |  |  |  |
| Poor outcome | 151 (64%) | 28 (38%) | 123 (75%) | <0.001 |
| 3-month mortality | 38 (16%) | 6 (8%) | 32 (20%) | 0.025 |
| 3-month mRS | 3.00 (2.00, 5.00) | 2.00 (1.00, 3.00) | 4.00 (3.00, 5.00) | <0.001 |
| ASPECTS reduction | 2.00 (1.00, 4.00) | 0.00 (0.00, 1.00) | 3.00 (2.00, 5.00) | <0.001 |
| sICH | 24 (10%) | 3 (4%) | 21 (13%) | 0.037 |

ASPECTS, alberta Stroke Program Early CT Score; AF, atrial fibrillation; PaCO_2_, partial pressure of arterial carbon dioxide; PaO_2_, partial pressure of arterial oxygen; pH, potential of hydrogen; SBP, systolic blood pressure; DBP, diastolic blood pressure; PP, pulse pressure; IQR, interquartile range; mRS, modified rankin score; NIHSS, national institutes of health stroke scale; sICH, symptomatic intracerebral hemorrhage; PLT, platelet. SIE, significant infarct enlargement.

**Supplementary Table III. Univariate and multivariate analysis of sICH in influencing factors (Logistic regression).**

|  | **Univariable** | | | | **Multivariable** | | |
| --- | --- | --- | --- | --- | --- | --- | --- |
| **Characteristic** | **OR** | **95% CI** | **p-value** | **OR** | | **95% CI** | **p-value** |
| Age | 1.03 | 1.00, 1.07 | 0.080 |  | |  |  |
| SEX(Female) | 1.14 | 0.47,2.67 | 0.763 |  | |  |  |
| Hypertension | 1.13 | 0.48, 2.90 | 0.785 |  | |  |  |
| Diabetes | 1.09 | 0.30, 3.11 | 0.881 |  | |  |  |
| AF | 1.47 | 0.63, 3.49 | 0.375 |  | |  |  |
| Smoking | - | - | 0.986 |  | |  |  |
| Stroke | 1.18 | 0.26, 3.77 | 0.801 |  | |  |  |
| PaCO_2_ | 0.96 | 0.87, 1.05 | 0.367 |  | |  |  |
| PaO_2_ | 1.00 | 0.99, 1.01 | 0.808 |  | |  |  |
| pH | 6.05 | 0.00, 213.73 | 0.738 |  | |  |  |
| High PaCO_2_ | 0.58 | 0.13, 1.79 | 0.398 | 0.67 | | 0.15, 2.14 | 0.540 |
| SBP (mmHg) | 1.00 | 0.98, 1.01 | 0.688 |  | |  |  |
| DBP (mmHg) | 0.99 | 0.96, 1.01 | 0.332 |  | |  |  |
| PP (mmHg) | 1.00 | 0.98, 1.03 | 0.744 |  | |  |  |
| Posterior circulation | 1.14 | 0.36, 3.03 | 0.808 |  | |  |  |
| NIHSS | 1.04 | 0.99, 1.09 | 0.159 |  | |  |  |
| Ischemic core volume | 1.01 | 1.00, 1.01 | 0.002 | 1.01 | | 1.00, 1.01 | 0.003 |
| Penumbra volume | 1.00 | 0.99, 1.00 | 0.444 |  | |  |  |
| IV thrombolysis | 2.31 | 0.99, 5.52 | 0.053 |  | |  |  |
| Angioplasty | 0.91 | 0.32, 2.30 | 0.853 |  | |  |  |
| PLT | 0.99 | 0.98, 1.00 | 0.040 | 0.99 | | 0.98, 1.00 | 0.052 |

OR, odds Ratio; CI, confidence interval; AF, atrial fibrillation; PaCO_2_, partial pressure of arterial carbon dioxide; PaO_2_, partial pressure of arterial oxygen; pH, potential of hydrogen; PP, pulse pressure; SBP, systolic blood pressure; DBP, diastolic blood pressure; IQR, interquartile range; NIHSS, national institutes of health stroke scale; sICH, symptomatic intracerebral hemorrhage; PLT, platelet.

**Supplementary Table IV. Univariate and multivariate analysis of 90-days mortality in influencing factors (Logistic regression).**

|  | **Univariable** | | | **Multivariable** | | |
| --- | --- | --- | --- | --- | --- | --- |
| **Characteristic** | **OR** | **95% CI** | **p-value** | **OR** | **95% CI** | **p-value** |
| Age | 1.03 | 1.00, 1.06 | 0.076 |  |  |  |
| SEX(Female) | 1.52 | 0.75, 3.06 | 0.240 |  |  |  |
| Hypertension | 2.85 | 1.26, 7.34 | 0.018 | 2.75 | 1.07, 8.07 | 0.047 |
| AF | 0.65 | 0.31, 1.32 | 0.241 |  |  |  |
| Diabetes | 3.79 | 1.69, 8.35 | 0.001 | 2.62 | 1.02, 6.56 | 0.041 |
| Smoking | 0.75 | 0.27, 1.79 | 0.539 |  |  |  |
| Previous diagnosis of stroke | 1.68 | 0.58, 4.29 | 0.304 |  |  |  |
| PACO_2_ | 0.92 | 0.85, 0.99 | 0.032 | 0.93 | 0.85, 1.02 | 0.128 |
| PAO_2_ | 1.00 | 0.99, 1.00 | 0.177 |  |  |  |
| pH | 0.47 | 0.00, 282.13 | 0.867 |  |  |  |
| High PaCO_2_ | 0.32 | 0.07, 0.95 | 0.069 |  |  |  |
| SBP (mmHg) | 1.00 | 0.99, 1.02 | 0.867 |  |  |  |
| DBP (mmHg) | 0.98 | 0.96, 1.00 | 0.074 |  |  |  |
| PP (mmHg) | 1.02 | 1.00, 1.03 | 0.081 |  |  |  |
| Posterior Circulation | 4.94 | 2.32, 10.55 | <0.001 | 3.55 | 1.19, 10.41 | 0.021 |
| NIHSS | 1.11 | 1.07, 1.16 | <0.001 | 1.07 | 1.01, 1.13 | 0.019 |
| Ischemic Core Volume | 1.01 | 1.00, 1.01 | 0.036 | 1.01 | 1.00, 1.01 | 0.011 |
| Penumbra Volume | 1.00 | 0.99, 1.00 | 0.953 |  |  |  |
| IV Thrombolysis | 1.77 | 0.87, 3.58 | 0.109 |  |  |  |
| Angioplasty | 0.83 | 0.35, 1.81 | 0.659 |  |  |  |
| PLT | 1.00 | 0.99, 1.00 | 0.710 |  |  |  |
| OR, odds ratio; CI, confidence interval; AF, atrial fibrillation; PaCO_2_, partial pressure of arterial carbon dioxide; PaO_2_, partial pressure of arterial oxygen; pH, potential of hydrogen; PP, pulse pressure; SBP, systolic blood pressure; DBP, diastolic blood pressure; PP: pulse pressure; IQR, interquartile range; NIHSS, national institutes of health stroke scale; PLT, platelet. | | | | | | |
|  | | | | | | |

**Supplementary Table V. Comparisons for baseline characteristics and outcomes between anterior circulation and posterior circulation.**

| Characteristic | Anterior circulation, N = 192 | | | | Posterior circulation, N = 45 | | | |  |
| --- | --- | --- | --- | --- | --- | --- | --- | --- | --- |
|  | Overall,  N = 192^1^ | Low PaCO_2_,  N = 152^1^ | High PaCO_2_,  N = 40^1^ | p-value^2^ | Overall,  N = 45^1^ | Low PaCO2,  N = 40^1^ | High PaCO_2_,  N = 5^1^ | p-value^3^ | Overallp-value^2^ |
| Age | 71 (61, 80) | 73 (63, 81) | 65 (57, 74) | 0.004 | 64 (57, 75) | 65 (56, 78) | 63 (61, 74) | 0.957 | 0.036 |
| Female | 76 (40%) | 72 (47%) | 4 (10%) | <0.001 | 16 (36%) | 16 (40%) | 0 (0%) | 0.144 | 0.618 |
| **Risk factors** |  |  |  |  |  |  |  |  |  |
| Hypertension | 123 (64%) | 100 (66%) | 23 (58%) | 0.331 | 29 (64%) | 26 (65%) | 3 (60%) | >0.999 | 0.962 |
| Diabetes | 27 (14%) | 24 (16%) | 3 (8%) | 0.180 | 10 (22%) | 9 (23%) | 1 (20%) | >0.999 | 0.175 |
| AF | 97 (51%) | 87 (57%) | 10 (25%) | <0.001 | 11 (24%) | 11 (28%) | 0 (0%) | 0.313 | 0.002 |
| Smoking | 38 (20%) | 19 (13%) | 19 (48%) | <0.001 | 8 (18%) | 7 (18%) | 1 (20%) | >0.999 | 0.759 |
| stroke | 19 (10%) | 16 (11%) | 3 (8%) | 0.769 | 7 (16%) | 6 (15%) | 1 (20%) | >0.999 | 0.291 |
| **Baseline characteristics** |  |  |  |  |  |  |  |  |  |
| PaCO_2_ | 36.0 ± 4.9 | 34.1 ± 3.5 | 43.2 ± 2.5 | <0.001 | 34.1 ± 4.3 | 33.2 ± 3.5 | 41.7 ± 1.0 | <0.001 | 0.013 |
| Severe low PaCO_2_ | 24 (13%) | 24 (16%) | 0 (0%) | 0.007 | 7 (16%) | 7 (18%) | 0 (0%) | 0.577 | 0.584 |
| PaO_2_ | 169 (137, 212) | 167 (136, 206) | 199 (147, 224) | 0.102 | 161 (137, 203) | 159 (137, 199) | 203 (193, 239) | 0.125 | 0.768 |
| pH | 7.40 ± 0.04 | 7.41 ± 0.04 | 7.36 ± 0.03 | <0.001 | 7.41 ± 0.04 | 7.42 ± 0.04 | 7.36 ± 0.02 | <0.001 | 0.024 |
| NIHSS | 17 (13, 20) | 17 (14, 20) | 17 (13, 20) | 0.906 | 31 (24, 35) | 31 (24, 35) | 24 (19, 25) | 0.180 | <0.001 |
| Ischemic Core Volume | 25 (11, 57) | 25 (12, 60) | 19 (8, 37) | 0.247 | 4 (1, 17) | 4 (1, 18) | 2 (1, 17) | >0.999 | <0.001 |
| Penumbra Volume | 92 (59, 132) | 93 (58, 132) | 92 (62, 137) | 0.872 | 82 (49, 113) | 82 (47, 113) | 83 (65, 109) | >0.999 | 0.203 |
| SBP (mmHg) | 156 ± 26 | 156 ± 26 | 153 ± 27 | 0.404 | 158 ± 19 | 159 ± 19 | 154 ± 23 | 0.661 | 0.455 |
| DBP (mmHg) | 88 (76, 101) | 90 (76, 102) | 86 (76, 94) | 0.231 | 86 (79, 100) | 87 (79, 99) | 82 (80, 100) | >0.999 | 0.942 |
| PP (mmHg) | 66 ± 20 | 66 ± 21 | 66 ± 20 | 0.944 | 70 ± 17 | 70 ± 18 | 65 ± 15 | 0.524 | 0.254 |
| PLT | 174 (138, 203) | 175 (133, 202) | 174 (148, 209) | 0.345 | 192 (138, 238) | 189 (130, 233) | 193 (147, 238) | 0.691 | 0.111 |
| Angioplasty | 42 (22%) | 29 (19%) | 13 (33%) | 0.068 | 21 (47%) | 19 (48%) | 2 (40%) | >0.999 | <0.001 |
| IV Thrombolysis | 66 (34%) | 58 (38%) | 8 (20%) | 0.031 | 19 (42%) | 16 (40%) | 3 (60%) | 0.636 | 0.323 |
| **Outcome** |  |  |  |  |  |  |  |  |  |
| Poor outcome | 115 (60%) | 99 (65%) | 16 (40%) | 0.004 | 36 (80%) | 33 (83%) | 3 (60%) | 0.258 | 0.012 |
| 3-month mortality | 21 (11%) | 20 (13%) | 1 (3%) | 0.083 | 17 (38%) | 15 (38%) | 2 (40%) | >0.999 | <0.001 |
| 3M-mRS | 3.00 (2.00, 4.00) | 3.00 (2.00, 5.00) | 2.00 (1.00, 4.00) | 0.004 | 5.00 (3.00, 6.00) | 5.00 (3.00, 6.00) | 4.00 (1.00, 6.00) | 0.563 | <0.001 |
| ASPECTS reduction | 2.00 (1.00, 4.00) | 2.00 (1.00, 4.00) | 1.00 (0.75, 3.00) | 0.013 | 3.00 (2.00, 5.00) | 3.00 (2.00, 5.00) | 3.00 (3.00, 8.00) | 0.454 | 0.007 |
| SIE | 126 (66%) | 107 (70%) | 19 (48%) | 0.007 | 37 (82%) | 33 (83%) | 4 (80%) | >0.999 | 0.031 |
| sICH | 19 (10%) | 17 (11%) | 2 (5%) | 0.373 | 5 (11%) | 4 (10%) | 1 (20%) | 0.461 | 0.786 |
| ^1^Median (IQR); n (%); Mean ± SD | | | | | | | | |  |
| ^2^Wilcoxon rank sum test; Pearson's Chi-squared test; Fisher's exact test; Welch Two Sample t-test | | | | | | | | |  |
| ^3^Wilcoxon rank sum test; Fisher's exact test; Welch Two Sample t-test  ASPECTS, alberta stroke program early CT score; PaCO_2_, partial pressure of arterial carbon dioxide; PaO_2_, partial pressure of arterial oxygen; pH, potential of hydrogen; PP, pulse pressure; AF, atrial fibrillation; SBP, systolic blood pressure; DBP, diastolic blood pressure; IQR, interquartile range; mRS, modified rankin score; NIHSS, national institutes of health stroke scale; sICH, symptomatic intracerebral hemorrhage; PLT, platelet. SIE, significant infarct enlargement. | | | | | | | | |  |
